# CRISPR arrays as high-resolution markers to track microbial transmission during influenza infection

**DOI:** 10.1186/s40168-023-01568-0

**Published:** 2023-06-17

**Authors:** Lingdi Zhang, Jahan Rahman, Matthew Chung, Lauren Lashua, Aubree Gordon, Angel Balmaseda, Guillermina Kuan, Richard Bonneau, Elodie Ghedin

**Affiliations:** 1 Department of Biology, Center for Genomics and Systems Biology, New York University, New York, NY 10003 USA; 2 Systems Genomics Section, Laboratory of Parasitic Diseases, National Institutes of Health, NIH, Bethesda, MD 20894 USA; 3 Department of Epidemiology, School of Public Health, University of Michigan, Ann Arbor, MI 48109 USA; 4 Sustainable Sciences Institute, Managua, Nicaragua; 5 Laboratorio Nacional de Virología, Centro Nacional de Diagnóstico Y Referencia, Ministry of Health, Managua, Nicaragua; 6 Centro de Salud Sócrates Flores Vivas, Ministry of Health, Managua, Nicaragua

**Keywords:** Metagenomics, Influenza virus, Microbiome

## Abstract

**Background:**

Disruption of the microbial community in the respiratory tract due to infections, like influenza, could impact transmission of bacterial pathogens. Using samples from a household study, we determined whether metagenomic-type analyses of the microbiome provide the resolution necessary to track transmission of airway bacteria. Microbiome studies have shown that the microbial community across various body sites tends to be more similar between individuals who cohabit in the same household than between individuals from different households. We tested whether there was increased sharing of bacteria from the airways within households with influenza infections as compared to control households with no influenza.

**Results:**

We obtained 221 respiratory samples that were collected from 54 individuals at 4 to 5 time points across 10 households, with and without influenza infection, in Managua, Nicaragua. From these samples, we generated metagenomic (whole genome shotgun sequencing) datasets to profile microbial taxonomy. Overall, specific bacteria and phages were differentially abundant between influenza positive households and control (no influenza infection) households, with bacteria like *Rothia*, and phages like *Staphylococcus P68virus* that were significantly enriched in the influenza-positive households. We identified CRISPR spacers detected in the metagenomic sequence reads and used these to track bacteria transmission within and across households. We observed a clear sharing of bacterial commensals and pathobionts, such as *Rothia*, *Neisseria*, and *Prevotella*, within and between households. However, due to the relatively small number of households in our study, we could not determine if there was a correlation between increased bacterial transmission and influenza infection.

**Conclusion:**

We observed that airway microbial composition differences across households were associated with what appeared to be different susceptibility to influenza infection. We also demonstrate that CRISPR spacers from the whole microbial community can be used as markers to study bacterial transmission between individuals. Although additional evidence is needed to study transmission of specific bacterial strains, we observed sharing of respiratory commensals and pathobionts within and across households.

Video Abstract

**Supplementary Information:**

The online version contains supplementary material available at 10.1186/s40168-023-01568-0.

## Introduction

Influenza infection as a contagious respiratory illness causes significant morbidity and mortality worldwide. Bacterial co-infection during influenza infection, particularly in the elderly and immunocompromised populations, can play an important role in disease progression leading to complications and severe disease outcomes [1]. Infections with respiratory viruses can also disrupt the microbiome of the airways and potentially contribute to disease severity [2]. Several studies have demonstrated viral disruption of the microbiota in the respiratory tract with changes in relative abundance of bacterial taxa such as *Pseudomonas*, *Corynebacterium*, and *Streptococcus* [3, 4].

Across body sites, such as the gut and skin, individuals from the same households have a more similar microbiome than individuals who do not cohabit [5, 6]. This apparent sharing of the microbiota can be due to various factors, including diet and genetics; however, direct bacterial transmission could also be a factor. For bacteria to transmit to a new host, the invading bacteria need to interact with the residing microbes and establish colonization [7, 8], which is more likely to occur when the microbiome in the new host is disrupted. The transmission of opportunistic pathogens in the respiratory tract, such as *Streptococcus pneumoniae*, is known to be associated with respiratory tract viral infection and younger age of the infected subject [7, 9]. Thus, we set out to study bacterial transmission in the respiratory tract in the context of influenza infection, which may disrupt the microbiome, further facilitating transmission.

Currently, most studies on bacterial transmission focus on specific bacterial species and use single-nucleotide polymorphisms (SNPs) in marker genes [10] or whole bacterial genomes [11, 12]. If using metagenomics data, this targeted approach would require very large sequencing depth and could only sufficiently profile SNPs from the most well-assembled bacterial genomes. An alternative is to focus on CRISPR arrays, which have been used in tracking specific bacterial strains from isolates [13, 14] as well as strain tracking and sequence diversity analysis from microbial communities [15, 16]. Here, we leveraged this unique nature of bacterial CRISPR arrays as markers to track transmission of bacterial communities within households during influenza infection.

CRISPR functions as the bacterial immune system to defend against virus infection by integrating a 20–70 bp viral spacer into the CRISPR locus when the bacteria are first exposed to the virus. Bacteria that have the integrated sequences are then able to defend themselves against viruses that match those spacer sequences [17]. Viral spacers are constantly acquired by the bacteria and integrated at the end of CRISPR arrays, proximal to the leader sequence [17]. Although the spacer sequences that the bacteria acquire from a specific virus are not entirely random, as bias in spacer sequence distribution has been observed [18, 19], the possible number of unique spacer sequences bacteria can acquire from a virus infection is large and thus random [20]. Given the dynamics of the CRISPR arrays, we demonstrate that these can indeed be used to specifically identify shared bacteria between the respiratory microbiome of different individuals, thus allowing us to leverage metagenomics datasets to potentially track the transmission dynamics of pathogens.

## Results

### Study cohort and sample collection

We obtained 221 respiratory samples (pooled nasal and throat swabs) that were collected from 54 individuals participating in the Household Influenza Transmission Study (HITS) in Managua, Nicaragua. In total, 10 households with 4–8 members in each household participated in the study, and samples were collected at 4 to 5 time points for each individual, at 2- to 4-day intervals. Sample collection was independent of influenza infection; thus, some of the samples were collected at time points when the individual was not yet infected or had recovered (Table S1). The households were assigned to high, low, or no influenza virus (control) infection groups based on the number of individuals per household who tested positive for influenza. High infection households had all or 2/3 of the household members testing positive at some point over the serial sampling (58 household members), while the low infection households had less than a third of household members testing positive for influenza at any time point (2–3 members). The “no flu” households represent uninfected controls (Table S1). We did not sample all the household members from the low influenza and control households. Influenza infection was diagnosed by rtPCR, and the infections were all due to influenza A virus subtype H3N2. Total DNA was extracted from each sample and was subjected to whole genome shotgun (metagenomics) for an in-depth microbiome analysis of the upper respiratory tract across household members. Of the 221 samples, we obtained 167 metagenomics datasets. Figure S1 provides an overview of the overall bioinformatics pipeline for this study with the different analyses performed.

### Microbial compositional differences between flu infection households

**Fig. 1:**
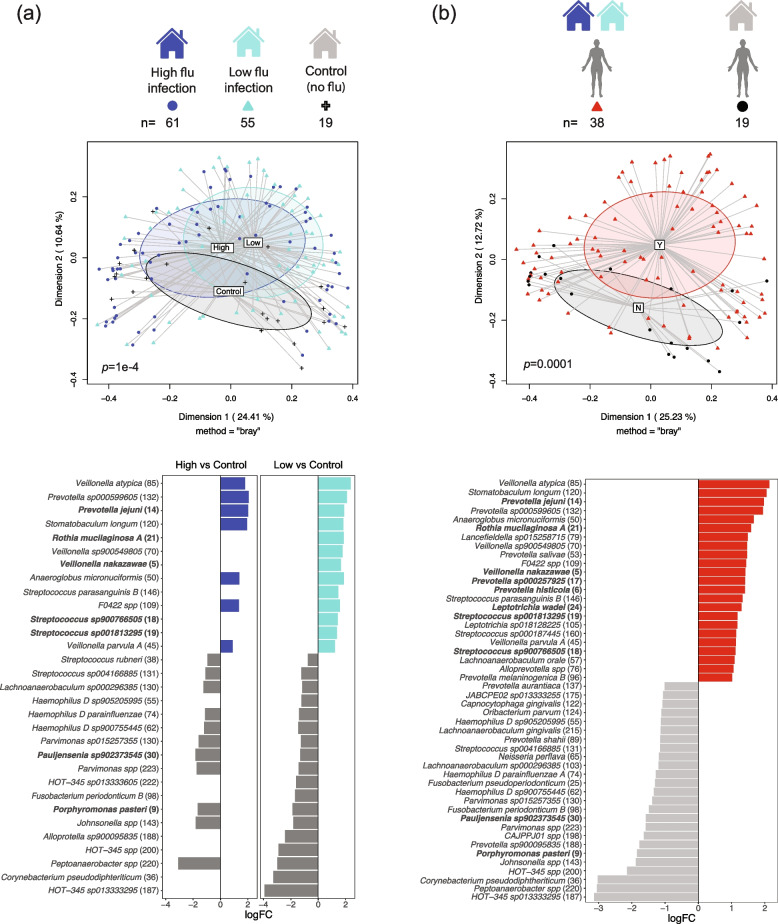
Differential taxa enrichment between individuals from flu infection and no infection households. **a** PCA plot of diversity of the microbial composition for different influenza infection households. Blue indicates high flu infection households, turquoise indicates low flu infection households, and gray indicates control households. Differential abundance of bacteria between high flu infection versus no flu infection or between low flu infection versus no flu infection households. **b** PCA plot of diversity of the microbial composition for flu-negative individuals from flu infection or control households. Red indicates flu infection households, and gray indicates control households. Differential abundance of bacteria between uninfected individuals from flu infection and control households. Numbers next to bacterial taxa names indicate relative abundance ranking across all samples; taxa in bold are part of the top 30 most abundant taxa (see Fig. S2)

To assess the quality of the data, we profiled the microbial composition in subjects across flu infection and control (no flu infection) households, as previous studies established that influenza can disrupt the microbial community, impacting diversity and composition of the microbiota [3, 4]. We first quality filtered the sequence reads and removed human reads from the datasets (median 6.8 M (*IQR* = 9.8 M), post filtering of human reads). We then assembled the reads into contigs to generate metagenome-assembled genomes (MAGs) and assessed bacterial origins of the MAGs by taxonomic assignment (Fig. S1a). Secondly, filtered reads were mapped back to each MAG to generate bacterial profiles for each sample (Fig. S1a). To analyze microbial communities across the household groups, we compared the relative abundance of bacteria for different comparisons (household subjects and samples are summarized in Tables 1 and 2). As there were more children (age < 18 years old) than adults with influenza infection in this cohort, we added age as a covariate in our analyses. We identified significant differences in bacterial diversity between household groups (PERMANOVA [21] *p*-value = 1e-4; Fig. 1a). We applied differential abundance analysis (limma [22]) to identify specific bacterial taxa that drove the differences in the beta diversity. We established that respiratory tract commensals and pathobionts such as *Rothia*, *Veillonella*, and *Prevotella* were significantly enriched in the high and low flu infection households, while *Haemophilus* and *Corynebacterium* were enriched in the no flu infection households (Fig. 1a). Some of the bacterial species that are differentially abundant between household groups are also ranked in the top 30 across samples for mean relative abundance (Fig. S2).

**Table 1:** Summary table for individuals across the households

	Flu infection			
Number of households	High infection *N* = 4	Low infection *N* = 4	No infection *N* = 2	*p*-values
Number of individuals	23	23	6	
Age median (SD)	8 (6.5)	13 (9.5)	7 (8.3)	0.0981
Gender				1
Female (%)	16 (70)	17 (70)	4 (70)	
Male (%)	7 (30)	6 (30)	2 (30)	

The *p*-values were calculated by using ANOVA and Fisher’s exact tests

**Table 2:** Summary table for flu infection and no infection samples

	Flu infection		
	Infection *N* = 42	No infection *N* = 93	*p*-values
Age median (SD)	6 (5.4)	14 (8.2)	4.47E-07
Gender			0.02051
Female (%)	15 (47)	74 (80)	
Male (%)	17 (53)	19 (20)	

The *p*-values were calculated using ANOVA and Fisher’s exact tests

By comparing flu-negative samples from individuals in the influenza infection households with the (flu negative) samples from the control households, we identified significant differences in microbial composition (*p*-value = 0.0001). Many of the bacteria enriched in the flu infection households (Fig. 1a) are also enriched in the flu-negative samples from the flu infection households (Fig. 1b) including pathobionts such as *Rothia*, regardless of influenza infection status.

To also identify phage origins for the MAGs, we used VirSorter [23] and vConTACT2 [24] and generated phage profiles with number of reads mapped to each viral MAGs per sample (Fig. S3). Across the dataset, the most prevalent MAGs identified by vConTACT2 cluster with *Chivirus* and Lily virus, which include *Salmonella* phage species and a phage of soil and insect bacteria, respectively (Fig. S3a). By comparing the relative abundance of these MAGs between household groups, we found MAGs clustering with the genera *P68virus* to be enriched in the high infection households (*FDR* = 0.042) and the ones clustering with *Triavirus* to be enriched in the low infection households (*FDR* = 0.043). These MAG clusters included *Streptococcus* and *Staphylococcus* phages (Fig. S3b). Top virus MAGs that clustered with phage genera, *Decurrovirus* and *Poushouvirus*, of common soil (*Arthrobacter*) and skin (*Corynebacterium*) bacteria, were enriched in the no infection households (*FDR* = 1.26e-5 and 0.043) (Fig. S3b).

### Shared CRISPR spacers to identify transmission events

**Fig. 2:**
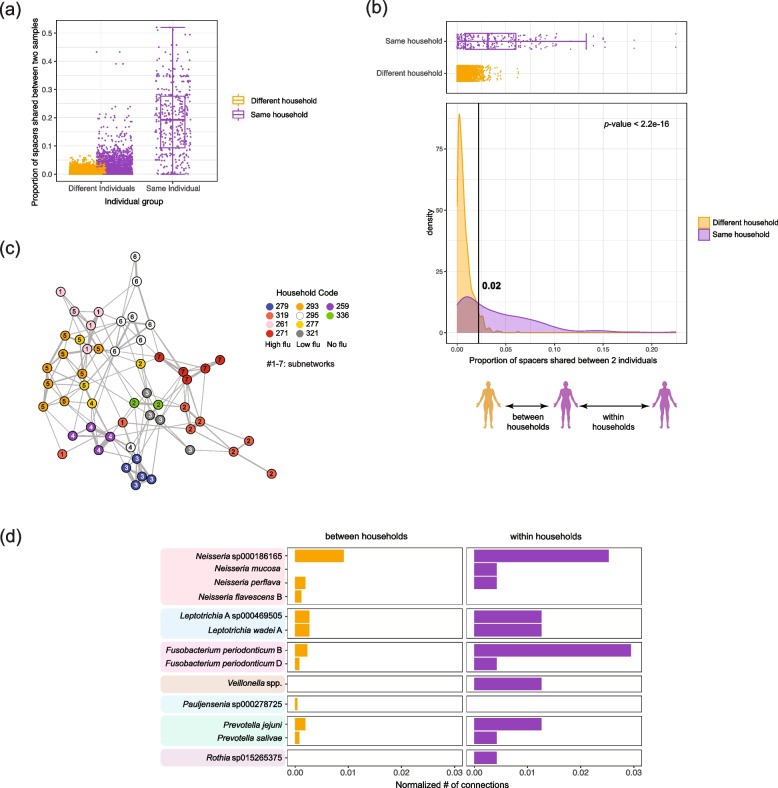
CRISPR spacers shared between samples and individuals. Proportion of shared spacers between samples or individuals were compared and used to construct the connection network between individuals. **a** Boxplot indicating proportion of spacers shared between samples from the same individuals, different individuals in the same households, and individuals from different households. The colors indicate whether the samples are from the same households (purple) or different households (orange). **b** Density plot and boxplot for proportion of spacers shared at the individual level within and between households. The black line on the density plot indicates the cutoff where there is the same number of comparisons within and between households. **c** The connection network was generated based on the proportion of shared spacers between individuals for the data above the cutoff in (**b**). The nodes represent individuals, and the edges represent proportion of shared spacers. Same color nodes indicate individuals come from the same household, and the numbers on the nodes represent the subnetwork they were partitioned into. **d** The barplots show the shared bacteria between individuals, normalized by the number of connections, and whether the individuals were from the same or different households

One important question when considering the disruption of the respiratory microbiome in a respiratory viral infection is whether certain bacteria with pathogenic potential are likely to be transmitted. Since many commensals and pathobionts are natural members of the respiratory community [25, 26], determining the dynamics of respiratory commensal bacteria shared within and between households is challenging when using metagenomic data. We thus used CRISPR spacers identified from the metagenomics data as potential barcodes for tracking bacterial transmission. Although CRISPR arrays have previously been used for strain identification of transmitted bacterial isolates [13, 14], we applied a similar approach but to track bacterial transmission within and across households from the respiratory metagenomes. We first identified spacer sequences from the metagenomics reads using Crass [27] where we found 188,876 spacers in total (Fig. S1b). We pooled the spacers across all the samples and identified spacers shared between samples based on 90% sequence similarity (61% of the spacers were unique; Fig. S1b). We then determined the proportion of spacers that were shared between any two samples. Samples from the same individual collected at different time points shared more spacers than samples from different individuals (Fig. 2a). We also found that the proportion of shared spacers was higher when comparing samples from individuals living in the same household and individuals from different households (Fig. 2a), indicating potential transmission within households. To further compare spacers identified from individuals within and across households, we pooled the serial samples for each subject and redid the analysis in subject-to-subject comparisons. Individuals from the same households have a higher proportion of shared spacers than individuals from different households (Fig. 2b), indicating more shared bacteria. As the number of subject-to-subject comparisons is not balanced for within and between households, we removed comparisons between individuals with lower than 2% shared spacers (Fig. 2b), leading to an equal number of comparisons within and between households, helping us weigh comparisons between individuals from the same and different households equally and removing noise. A connection network was then generated based on the proportion of shared spacers between individuals (Fig. 2c) where the nodes are the individuals, and the edges are weighted by the value of the proportion of shared spacers. We also detected subnetworks within the network using the shared spacer data between individuals (Fig. 2c). The correlation between the partition of the nodes to the subnetworks and the household metadata was 0.79, indicating individuals within the same households were more tightly connected based on their proportion of shared spacers.

To determine which bacterial taxa were shared between individuals from the spacer profiles, we mapped the reads containing shared spacers to the bacterial MAGs for which we obtained taxonomic assignments (Fig. S1b). Given that the insertion of the phage sequences into the bacterial genomes is mostly random [20], the bacterial species that contain spacers that are the same in different individuals can be inferred as being shared between these individuals. We thus found bacterial species from 7 bacterial genera that we infer to be shared within and between households (Fig. 2d). The shared bacteria included respiratory commensals and pathobionts such as *Rothia* and *Neisseria*. We compared the enrichment of shared bacteria between household groups, but while some bacteria were shared more often within certain household groups than others (Fig. S4), we did not have power to get statistical support.

**Fig. 3:**
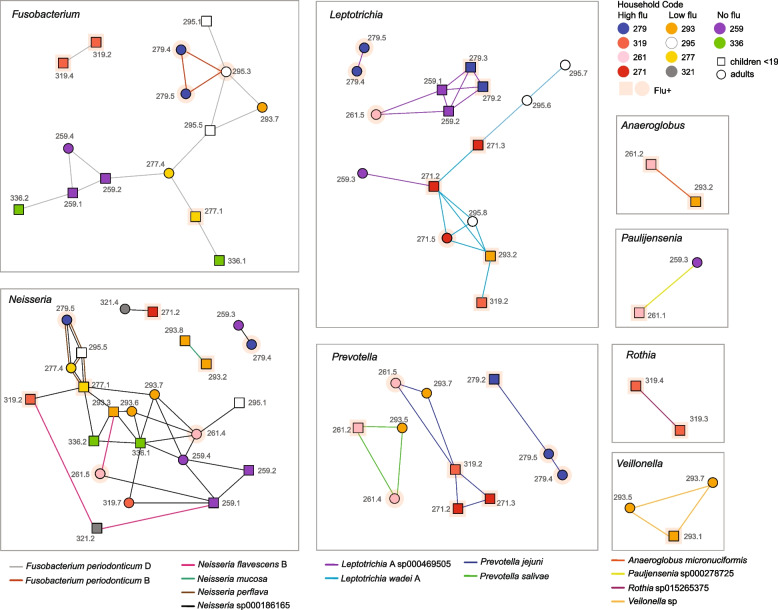
Network connecting individuals with shared bacteria. Individuals sharing the same bacteria strains identified by CRISPR spacers were linked with the subject IDs shown next to the nodes. The color of the nodes indicates household information. The bacteria taxa are shown in the legend and colors of the edges. Circle and box indicate whether the subjects are children or adults; pink color is to highlight flu-positive individuals

From the spacer analysis, we have observed more sharing of spacers between time points from the same individual than across individuals (Fig. 2a). However, there are also changes in the spacer contents across time points from the same individuals (median normalized connections between time points from the same individuals were 0.19, Fig. 2a). Thus, we further investigated the spacers mapped to the bacterial species we mentioned above (Fig. 2d) but for each time point individually (Fig. S5). We captured changes in CRISPR spacer content over time as there were shared spacers across time points, as well as unique spacers for the specific bacteria analyzed (Fig. S5). To evaluate how these bacteria were shared between individuals across our complete cohort, we constructed networks using the MAGs that have shared spacers (Fig. 3). In the shared bacterial networks, however, there was no overrepresentation of flu-infected individuals. Even when testing for overrepresentation of shared bacteria within flu infection households as compared to the control households and removing individual pairs that shared less than 6% of the spacers (Fig. S6a), thus focusing on individuals within the same households (Fig. S6b), there was no correlation between bacterial transmission and flu infection levels (Fig. S6c). We also compared the proportion of shared spacers across pairs of individuals when (a) both individuals were infected with flu, (b) one of them was infected, or (c) neither was infected (Fig. S7). Although we did get significant *p*-values, the differences in the distributions were moderate.

## Discussion

The respiratory tract microbiome, because of its function in health [25], should play an important role during respiratory tract infections. Here, we generated metagenomic datasets from nasal and throat swabs to profile bacterial taxa and track bacterial transmission within and across households. We observed that while influenza-positive households were significantly different in microbial composition from the control (flu negative) households, with a few bacteria differentially abundant between the groups, there was no significant difference in how bacteria were shared within households, with or without influenza.

Among the bacterial species enriched in the infection households, we identified species of the potentially pathogenic genus *Rothia* [28]. We also observe various *Streptococcus* species enriched in both the infection and control households, although some species of *Streptococcus* are known to associate with viral-bacterial co-infections [29–31]. Similarly enriched in the no infection households was *Corynebacterium*, which was previously found to be negatively associated with influenza infection [3]. Our study shows a household effect on the microbiome in influenza infection as we observed that individuals from flu infection and no infection households had different airway microbial profiles. This observation supports other studies showing that some individuals may be more vulnerable to influenza infection due to their microbiome composition differences [32], and that modulation of the microbiome could help protect against influenza infections [33].

Other studies have also observed microbiome compositional similarities between individuals within the same households [5, 6], such as for the skin microbiome due to similar exposure to external conditions [6]. However, analyses based on microbial compositional similarities cannot differentiate between the effects of diet, genetic inheritance, and transmission that shape the microbiome. In this study, we demonstrate potential transmission of respiratory bacteria that contribute to the shared microbiota within households. The use of CRISPR arrays to identify bacterial species and track bacterial transmission was previously done using bacterial isolates [13, 14] and metagenomics data type [15]. The novelty of our study is that we leverage for the first time CRISPR array spacers identified in the metagenomics data from clinical samples to track short-range bacterial transmission within and between households. We showed a higher percentage of shared CRISPR spacers between individuals from the same households, which is likely due to transmission events. The individuals connected with shared bacteria include both children and adults, with and without influenza. Children within households can drive bacterial transmission as they may have closer contact with other household members. However, we do not have bacterial isolates or longer time points before influenza infection to validate bacterial transmission and to determine whether this happened during influenza infection.

By using the CRISPR spacers and bacterial genomes that the shared spacers mapped to, we have shown shared bacterial strains within and across households. The dynamics of the CRISPR array contents are affected by various factors. Coevolution between the CRISPR system and the phages it encounters [19, 34], DNA recombination of the CRISPR arrays between bacterial strains [35], and the balance between maintaining the CRISPR systems and survival [36] can contribute to the evolution of the CRISPR arrays. Although we were not able to infer the evolutionary rates of the CRISPR arrays in our data due a lack of full-length CRISPR arrays for all MAGs, we showed that we could capture the dynamic nature in the CRISPR array contents for the shared bacteria across sampling timepoints. Thus, the CRISPR arrays of the shared bacteria we analyzed were actively replacing or incorporating spacers, at least within the time frame of our sample collection (9- to 12-day period of sample collection). Coupling these types of data with long-read sequencing datasets would likely allow better tracking of specific bacterial strain transmission on a larger scale.

There are a few limitations in this study. First, while the use of CRISPR arrays did allow the identification of shared bacteria between individuals, not all bacterial species have a CRISPR system [37]; thus, our analysis is restricted to a limited set of bacteria. Also, we do not have bacterial isolates paired with the metagenomics datasets, which would have allowed us to estimate CRISPR evolutionary rates for different bacterial species. Second, we were limited by the number of households in the study and thus cannot draw any conclusion between bacteria-sharing and influenza infection rate. The households with high or low influenza infection only indicate the members in the households were infected with influenza, but we do not have estimates of influenza infection patterns (i.e., who infected whom) within these households.

In conclusion, the analysis of the metagenome data demonstrates microbiome compositional differences between individuals from influenza infection and no infection households. Despite these differences, bacteria appear to be readily transmitted within and across households in both flu-positive and control individuals. We demonstrated CRISPR spacers can be used to study bacterial transmission in the microbial community using metagenomics datasets. However, although we showed commensal bacteria and potential pathobionts are shared within and across households, CRISPR array evolution rates are needed to validate specific bacterial transmission between individuals.

## Material and method

### Data collection

Samples were collected from individuals participating in the Household Influenza Transmission Study (HITS) in Managua, Nicaragua, between July 2013 and October 2014. The HITS sample cohort included child index cases enrolled in the Nicaraguan Influenza Cohort Study and their family members who developed influenza as well as some influenza-negative control households. Respiratory specimens consisted of pooled nasal and throat swabs collected from household members every 2–4 days over a 9- to 12-day period. Samples were shipped to the Center for Genomics and Systems Biology, New York University, and stored at – 80 °C. The HITS study was approved by the institutional review boards at the Nicaraguan Ministry of Health and the University of Michigan. Informed consent or parental permission was obtained for all participants, and children aged 6 years and older provided assent.

### DNA isolation and library preparation for metagenome sequencing

Genomic DNA was isolated from the remaining volume of each sample with the PowerSoil DNA Isolation Kit (Qiagen) and stored at − 80 °C. Libraries were generated using Nextera DNA Flex Library Prep Kit (Illumina, Inc., San Diego, CA, USA). Libraries were quantified by qPCR using the KAPA Library Quantification Kit (KAPA Biosystems, Wilmington, MA, USA) on a Roche 480 LightCycler (Roche, Basel, Switzerland); their size distributions were measured on a 4200 TapeStation using a D1000 ScreenTape (Agilent Technologies, Santa Clara, CA, USA). Libraries were diluted to 4 nM in dilution buffer (10-mM Tris, pH 8.5) and combined with equimolar input into 9 sequencing pools (20 − 25 libraries per pool). Paired-end sequencing (2 × 150 bp) was performed at the Genomics Core Facility (Center for Genomics and Systems Biology, New York University) on the Illumina NextSeq 500 instrument according to the manufacturer’s instructions (Illumina, Inc., San Diego, CA, USA) with a few libraries sequenced on the Illumina HiSeq 2500 instrument.

### Metagenomics data processing and bacterial taxonomic assignments

The metagenomics reads were filtered to remove adaptors and low-quality reads using Trimmomatic v0.36 [38] followed by DeconSeq2 v1.32.0 [39] to remove human reads. The median reads number for metagenomes was 6.8 M (*IQR* = 9.8 M).

### Bacterial and viral taxonomic assignments

Processed FASTQs were assembled into metagenome-assembled genomes (MAGs) using metaSPAdes v3.15.2 run with default settings [40]. For the identification of bacterial MAGs, the processed FASTQs were mapped to a catalogue of MAGs from all samples using minimap2 v2.24 [41] keeping at most 5 secondary alignments. Using vamb v3.0.9 [42] with a minimum bin size of 200 k bp, the MAG catalogue was used with mapping data to bin MAGs based on similarity and co-abundance information. GTDB-tk v2.1.1 [43] was used to assign taxonomic classifications to each of the bacterial MAG bins.

For the viral MAG analysis, VirSorter2 v2.2.3 [23] was used to identify phages in our MAG catalogue with a minimum length of 1-kbp run with the flag –keep-original-seq. The potential host regions left at the ends of the proviruses were trimmed from the identified phage contigs using checkV v0.8.1 [44]. Protein sequences were called for all viral sequences using prodigal v2.6.3 [45] with the flag -p meta. Protein sequences were used to taxonomically identify each viral contig using vConTACT2 v0.11.3 [24] against the viral RefSeq database using Diamond v0.9.24 [46] to create the protein–protein similarity matrix, MCL v14-137 [47] to generate protein clusters, and ClusterONE v1.0 [48] to generate viral clusters.

Counts for bacterial and viral MAGs were calculated by mapping the reads back to their respective MAG catalogues using minimap2 v2.24. Alignment files were filtered to exclude non-primary and secondary alignments using the SAM flag 2308. Read counts to each contig were quantified using the idxstats function of samtools v1.9 [49].

### Bacteria and virus differential abundance analyses

Beta diversity of the metagenomics datasets was determined using Bray–Curtis distance, and the global diversity between different groups was determined by PERMANOVA [21]. The bacterial taxa differential abundance analysis was done using DAtest (Version 2.8.0) where 21 differential abundance analysis methods were tested against the datasets. Because limma had the highest score, AUC, and power in the analysis, we ran limma on the bacterial taxonomic profiles and viral taxonomic profiles to identify bacteria differentially abundant between the groups with an FDR smaller than 0.05.

### CRISPR spacer analysis and network analysis

The spacers were identified from each metagenomics dataset using Crass [27]. The spacers across all the samples were clustered, and spacers with sequence similarity greater than 90% using CD-HIT [50] were determined as being the same spacers across samples. The percent of shared spacers between samples was determined as the number of shared spacers between any two samples divided by the average of total spacers in the two samples. Percent of shared spacers was compared between samples from the same individuals, different individuals in the same households, and different households. When comparing at the individual level, the spacers from different time points for the same individual were combined to do the analysis. The network connecting individuals based on shared spacers was generated using igraph [51] in R studio, and the edge weight was the percent of shared spacers between individuals. Subnetworks were also analyzed using igraph. Reads contain the spacers were mapped back to their respective MAG catalogues using minimap2 v2.24. Alignment files were filtered to exclude non-primary and secondary alignments using the SAM flag 2308.

## Supplementary Information

**Additional file 1: Fig. S1.** Analysis pipeline. (a) *(yellow and red paths)* The metagenomics reads post-quality filtering and removal of human reads were assembled into contig—metagenome assembled genomes (MAGs)—using metaSPAdes. Viral MAGs were identified using CheckV and VirSorter2 and taxonomic assignments were done using vConTACT2. Bacterial MAGs were binned with vamb and taxonomic assignment was done using GTDB-Tk. We then mapped the reads back to the taxonomically assigned viral or bacterial MAGs to generate the bacterial and viral profiles for downstream differential abundance analyses. (b) (*brown paths*) We identified and extracted the spacers from the metagenomic reads using Crass. Spacers with 90% sequence identity were clustered together. We then used these spacers to identify the shared spacers between individuals and within and across households. Reads with spacers shared between individuals were mapped to the bacterial MAGs that had taxonomic assignments. Bacterial species containing shared spacers were identified as being shared between individuals. **Fig. S2.** Top 30 most abundant bacterial taxa. Bacterial taxa were ranked by their mean relative abundance across samples. The top 30 bacterial taxa are shown in the boxplot with the x axis representing the relative abundance for each sample. **Fig. S3.** Viral MAGs identified. (a) Top viral MAG taxa identified as sorted by median relative abundance. The x-axis shows the relative abundance of the viral taxa of all samples while the y-axis indicates the viral taxa, listed as family/genus and phage species included within MAG clusters. (b) Viral MAGs differentially abundant between the high flu infection household vs control or low flu infection household vs control, identified with FDR cut-off as 0.05. The log2 fold changes are shown on the x axis with the blue/turquoise indicating flu infection household groups and gray the control household group. **Fig. S4.** Shared bacteria between flu infection households. The bar plots show the bacteria shared between individuals and how many pairs of individuals from the high flu infection, low flu infection and no flu infection households shared bacteria. **Fig. S5.** Mapping of the spacers to the bacteria shared between individuals for each sample. For all panels, the x axis represents the spacers mapped to the specific bacteria as indicated by the plot titles while the y axis represents the subject ID and timepoint of the sample. The colored dots mark households, as per Fig. 3. The shaded boxes indicate which family members had a direct connection based on the sharing of bacteria. **Fig. S6.** Sharing of Bacteria and flu infection. (a) Density and boxplot plot for percent of spacers shared at the individual level within and between households. The red line on the density plot indicates the cut-off where all the “between household” individual pairs were removed. (b) The connection network was generated based on the percent of shared spacers between individuals for the data above the cut-off in (a). The nodes represent individuals and the edges represent percent of shared spacers. Same color nodes represent individuals from the same household. (c) Dotplot for proportion of individuals in each household that were connected. Number of individuals in (b) in each household were divided by the total number of individuals in the households and compared across flu infection groups. The x axis indicates household code and the panels show the household from high, low, or no flu infection groups. **Fig. S7.** Proportion of shared spacers between individuals with different flu infection status. The box plot shows the proportion of shared spacers between any two individuals that were: (1) both positive for flu, (2) one positive for flu, one negative for flu or (3) both negative for flu. We compared the proportion of shared spacers between the three groups and Kruskal-Wallis test *p* values are shown between any two groups. * indicates *p* values <2.22e-16.

**Additional file 2: Table S1.** Sample metadata.

## Data Availability

The sequencing datasets supporting the conclusions of this article are available in the Sequence Read Archive (SRA). Metagenome data are under BioProject PRJNA713420. Scripts generating the data are available on GitHub (https://github.com/GhedinLab/Nicaragua_microbiome_flu_analysis).
