# Supplementary material for: CRISPR arrays as high-resolution markers to track microbial transmission during influenza infection
Source: Microbiome. 2023 Jun 17;11:136. doi: 10.1186/s40168-023-01568-0 (PMC10276449; doi:10.1186/s40168-023-01568-0)
Supplement: Supplementary file 2 — Additional file 1: Fig. S1. Analysis pipeline. (a) (yellow and red paths) The metagenomics reads post-quality filtering and removal of human reads were assembled into contig—metagenome assembled genomes (MAGs)—using metaSPAdes. Viral MAGs were identified using CheckV and VirSorter2 and taxonomic assignments were done using vConTACT2. Bacterial MAGs were binned with vamb and taxonomic assignment was done using GTDB-Tk. We then mapped the reads back to the taxonomically assigned viral or bacterial MAGs to generate the bacterial and viral profiles for downstream differential abundance analyses. (b) (brown paths) We identified and extracted the spacers from the metagenomic reads using Crass. Spacers with 90% sequence identity were clustered together. We then used these spacers to identify the shared spacers between individuals and within and across households. Reads with spacers shared between individuals were mapped to the bacterial MAGs that had taxonomic assignments. Bacterial species containing shared spacers were identified as being shared between individuals. Fig. S2. Top 30 most abundant bacterial taxa. Bacterial taxa were ranked by their mean relative abundance across samples. The top 30 bacterial taxa are shown in the boxplot with the x axis representing the relative abundance for each sample. Fig. S3. Viral MAGs identified. (a) Top viral MAG taxa identified as sorted by median relative abundance. The x-axis shows the relative abundance of the viral taxa of all samples while the y-axis indicates the viral taxa, listed as family/genus and phage species included within MAG clusters. (b) Viral MAGs differentially abundant between the high flu infection household vs control or low flu infection household vs control, identified with FDR cut-off as 0.05. The log2 fold changes are shown on the x axis with the blue/turquoise indicating flu infection household groups and gray the control household group. Fig. S4. Shared bacteria between flu infection households. The bar [file 40168_2023_1568_MOESM1_ESM.zip › 40168_2023_1568_MOESM1_ESM/FigS4_Zhang.pdf]

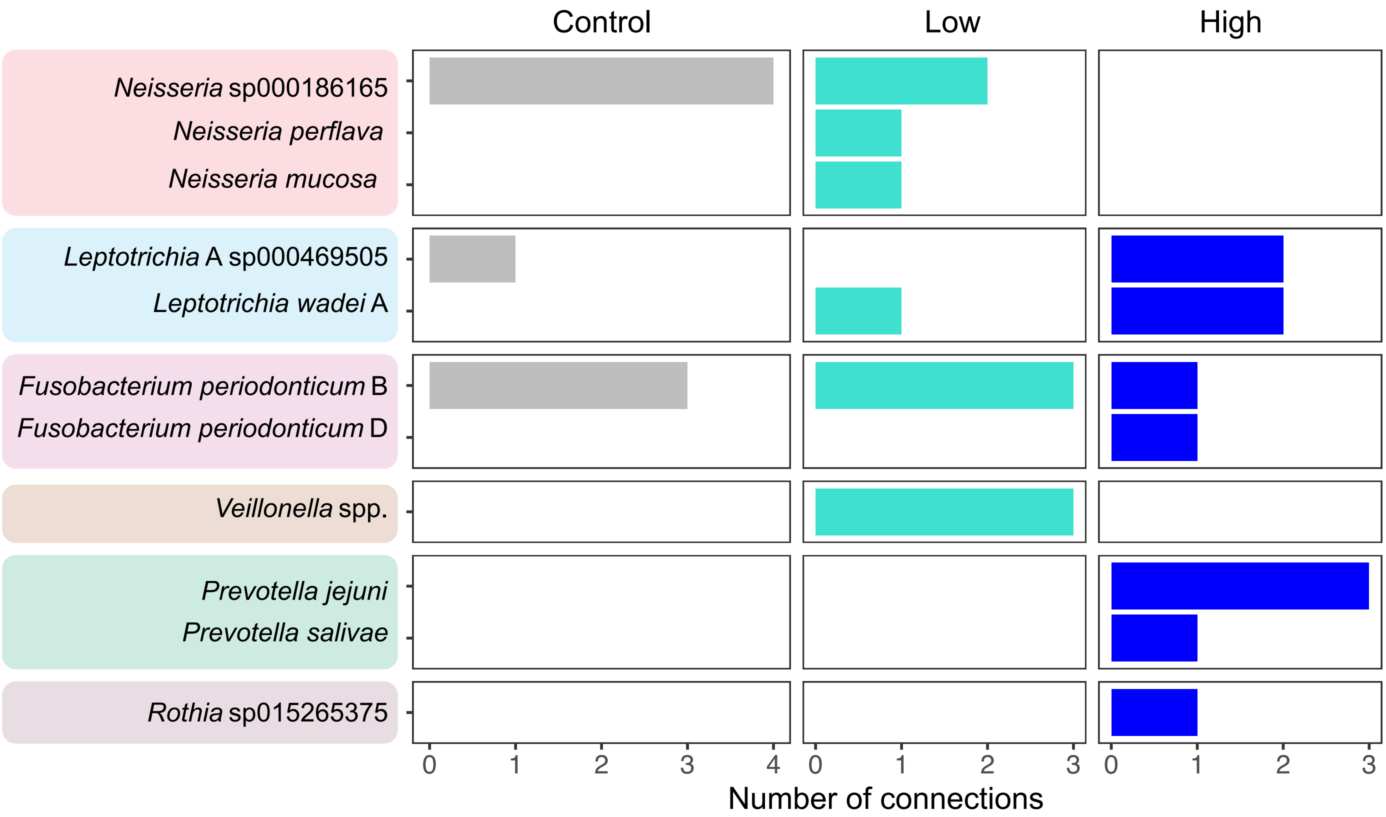

Figure S4. Shared bacteria between flu infection households. The bar plots show the bacteria shared between individuals and how many pairs of individuals from the high flu infection, low flu infection and no flu infection households shared bacteria.
